# Home-Based Telemedicine for Children with Medical Complexity

**DOI:** 10.1089/tmj.2018.0186

**Published:** 2019-11-06

**Authors:** Patricia M Notario, Elise Gentile, Matthew Amidon, Denise Angst, Cheryl Lefaiver, Kathleen Webster

**Affiliations:** ^1^ Billings Clinic, Billings, Montana.; ^2^ Advocate Children's Hospital, Oak Lawn, Illinois.; ^3^ Medical College of Wisconsin, Milwaukee, Wisconsin.; ^4^ Advocate Health Care, Downers Grove, Illinois.

**Keywords:** *telehealth*, *telemedicine*, *pediatrics*, *home health monitoring*

## Abstract

**Background:** Children with medical complexity (CMC) are high utilizers of health care services. Telehealth encounters may provide a means to improve care outcomes for this population.

**Objective:** To evaluate the feasibility, usability, and impact of an in-home telehealth device in the care of CMC.

**Methods:** This single-center feasibility study employed a nonblinded randomized clinical trial design. English-speaking caregivers of children within a pediatric complex care program with home Wi-Fi were eligible for participation. Participants were randomized 1.5:1 with stratification based on tracheostomy status to a control group that received usual care or an intervention group that received a telehealth device for in-home use. Patients were followed up for 4 months. The primary outcome was successful device connectivity and data transmission. Data included clinician encounter device usability; caregiver satisfaction; and encounter type, purpose, and cost. Descriptive statistics, negative binomial regression, and Kaplan–Meier plot were used for analysis.

**Results:** Twenty-four patients were enrolled (9 controls, 15 in the intervention group) in September 2016. The telehealth device was attempted in 73 encounters. Device connectivity was successful 96% of the time. Image and sound quality were acceptable in 98% of visits. Caregivers expressed their overall satisfaction with the device. The hospitalization rate was lower in the intervention group (0.77 vs. 1.14 intensive care unit days/patient-months), resulting in $9,425/USD per patient savings compared with the control group.

**Conclusion:** Despite small sample size and short observation period, this study demonstrated that use of an in-home telehealth device is feasible, well received by caregivers, and can result in decreased hospitalizations when compared with usual care.

## Introduction

Children with medical complexity (CMC) are high utilizers of health care services. Representing ∼5% of the U.S. pediatric population, they incur >60% of all children's health care expenditures.^1^ CMC often encounter multiple medical comorbidities, dependence on medical technology, severe functional limitations, financial strain for caregivers, and disjointed medical care.^2–4^ Transportation costs and logistics may result in delays in seeking care and evaluation in emergency departments (EDs).^5,6^ In addition, CMC are susceptible to sudden changes in physiologic status, frequently leading to utilization of hospital-based health care.^1,7^

Telehealth technology allows more complete remote encounters compared with telephone calls between patients and clinicians. Telehealth encounters may include the use of peripheral devices such as a stethoscope, otoscope, and camera that can provide data equal or superior to in-person examinations.^8–10^ A growing number of studies have shown feasibility, lower costs of care, and high clinician satisfaction when using telehealth in adult and pediatric populations.^8,11,12^ However, there are limited studies evaluating the use of telehealth in the care of CMC. Those published focus on the use of videoconferencing alone and demonstrated that videoconferencing increases family satisfaction, and decreases travel, ED, office visits, and hospitalizations for CMC.^13,14^

Few studies address the role of a remote examination device, and none describe the use of a device by a home caregiver of CMC. The objective of this study was to evaluate the feasibility, usability, and impact of a new telehealth device in home-based care of CMC within an existing pediatric complex care program. The primary outcomes of this study were feasibility, defined as successful connectivity with clinicians, and usability, the ability to complete a physical examination facilitated by caregivers, including transmitting real-time images, temperature, and sound. Secondary outcomes focused on the impact of the telehealth device, including changes in patient management, hospitalizations, family and clinician satisfaction, and costs associated with inpatient and outpatient care.

## Materials and Methods

### Participants

This study was designed as a single-center, randomized, nonblinded feasibility study. The sample size was determined by the number of eligible patients enrolled in our institution's pediatric complex care program. Inclusion criteria were as follows: age 1 month to 18 years, enrolled in the complex care program; parent consent; at least one English-speaking parent; and in-home Wi-Fi connectivity. Children within the complex care program met the following criteria: three or more body systems requiring active management; technology dependent or full support to complete activities of daily living; and moderate to severe neuromotor or intellectual disabilities. Children were excluded if caregivers expressed their inability to comply with study requirements. Participants were randomized 1.5:1 with stratification based on tracheostomy status to control group or intervention group (Research Randomizer, Urbaniak). This stratification was done based on the assumption that patients with tracheostomies may require more frequent contact with the care team. This study was approved by the Advocate Health Care Institutional Review Board and registered at ClinicalTrials.gov^*^

### Telehealth Device

**Fig. 1.**
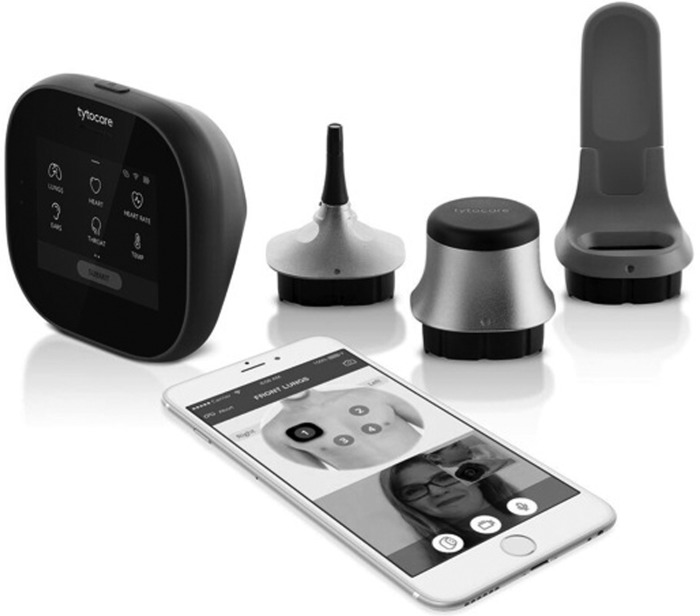
Tyto^™^ device setup and caregiver view. Image provided by TytoCare, Inc.

The selected telehealth device (TytoHome™) is an Food and Drug Administration cleared, handheld, mobile device designed for capture and transmission of ear, throat, and skin images; heart and lung auscultations, including heart rate detection; and temperature taken by infrared transdermal thermometer (*Fig. 1*). The device is paired with an iOS tablet (Apple iPad^®^ mini 4) for wireless network transmission of a live-interactive connection. Caregivers used the device to facilitate noninvasive medical examinations in the home guided by a remote clinician.

### Procedures

Enrolled caregivers provided informed consent during a home visit before study initiation. For intervention group patients, members of the TytoCare^™^ team accompanied the study team to explain use of the telehealth device and iPad mini. Home internet connectivity was assessed and supplemented if needed.

All caregivers were instructed to contact the clinician by telephone or e-mail as usual with health concerns. If an examination was deemed necessary by the clinician, control group patients were referred for an in-person encounter, whereas caregivers of patients in the intervention group were directed to connect through the telehealth device. Subsequently, the clinician conducted a two-way, live, interactive audio/video visit with the patient. As clinically indicated, the clinician would direct the caregiver to use the telehealth device to provide temperature, lung sounds, heart sounds, oropharyngeal examination, skin examination, and/or ear examination. For all patients, the clinician would direct treatment or refer to an ED, clinic visit or hospitalization based on available data. The intervention group also had scheduled telehealth visits for routine care such as postdischarge care and follow-up for a particular concern. In addition, due to technology issues encountered in the first month of the study, caregivers were contacted by the study team every 2–4 weeks throughout the study to help caregivers maintain familiarity with the telehealth device (practice visits).

### Data Collection

Technology issues resulting in failed connections were encountered during the first month of the study. The device had not previously been used in the home environment. Investigation revealed an issue requiring a technical modification to the device software. Since the software was modified, the postmodification encounters were deemed unequal to those premodification. To collect the intended 3 months of data on device use, the study period was extended, and data collection for the study period began after the device modification. The protocol modification was submitted to and approved by the IRB. The premodification period data are reported separately. Data collection throughout all 4 months included subject demographics, encounter details (outpatient clinic visits, ED visits, and hospitalizations), as well as caregiver and clinician surveys, which used a 4-point Likert response scale. All caregivers answered questions about complex care program satisfaction, and those in the intervention group answered additional questions regarding the device. Caregivers were e-mailed a link to the online survey once a month during the study period. Surveys, not completed online, were conducted through telephone by a member of the study team who did not participate in clinical care. Clinicians completed an online survey for each encounter in which they would have desired a telemedicine visit regardless of child's group assignment. Specific measures (*Table 1*) included general satisfaction, demographics, success of device connection, as well as transmission of real-time images, temperature, and sound. Potential or actual changes in patient management resulting from the telehealth examination were recorded.

**Table 1.** Study Aims and Measures

AIM	MEASURES	GROUPS
1. Feasibility	Clinician survey—questions about success of connection	Intervention
2. Usability	Clinician survey—questions about quality of telehealth assessment and user satisfaction	Intervention
	Parent survey	Intervention and control
3. Impact	Clinician survey—data gathering ability, device potential	Intervention and control
	Complex care program encounter tracking—encounter disposition; total number of encounters; resource utilization	Intervention and control

### Data Analysis

Descriptive statistics were used to summarize data with absolute and relative frequencies for categorical variables and means and standard deviations for continuous variables. Feasibility and usability of the device were determined from survey responses, and reported as median and interquartile range (IQR). Continuous variables were examined using independent groups Student's *t* test or the Mann–Whitney *U* test depending upon normality of data. Dichotomous variables were examined using chi-square or Fisher's exact test. Data are reported for the premodification month and subsequent 3-month observation period. Of note, if a clinical concern was identified during a practice visit, the visit was reclassified as an unscheduled telehealth visit.

The impact of device utilization was evaluated by measuring the total number of visits per patient by type of visit (outpatient; ED without subsequent admission, general pediatric ward, pediatric intensive care unit [ICU]) as determined through chart review. To adjust for the difference in sample size per group, the number of hospitalization days, acute outpatient visits, and ED visits were reported descriptively as a *visit rate* calculated as the number of visits or hospital days/patient study months (number of patients in the group × number of study months). The Kaplan–Meier method was used to estimate the proportion of each group that did not have any hospitalizations during the study period. A statistical comparison of the survival distribution was not conducted because the Kaplan–Meier method does not account for repeated episodes such as patients with more than one hospitalization.

A negative binomial regression model with generalized estimating equations to handle repeated measurements was used to test the difference in the length of stay in hospital between groups. Model results were reported as regression coefficients, 95% Wald confidence intervals, and *p*-values. The negative binomial regression model was appropriate for this analysis, as the distribution of the outcome had greater variability than expected under a Poisson distribution. The sample mean of the outcome (4.3) was substantially smaller than its variance (102.3). Regression models were not tested for the number of acute office or ED visits because the incidence rate was too small for the inferential statistic. Analyses were conducted using SPSS (IBM SPSS Statistics for Windows, version 22.0, Released 2013; IBM Corp. Armonk, NY). For all analyses, a *p*-value <0.05 was considered statistically significant.

Direct cost for each encounter was obtained from our institution's financial accounting system (EPSi), which was used to calculate direct costs per study group and encounter type (not including overhead costs such as cost of the telehealth device or connectivity). For the intervention group, a telemedicine visit was equated to a Level 4 return encounter in the outpatient setting. Direct cost savings were calculated as the absolute difference between the two study groups. To adjust for the difference in group size, the encounter direct cost was multiplied by the visit rate, and the calculated cost rates were reported for each group. Potential cost savings were calculated using clinician input on the visit types that were likely prevented as a result of the telemedicine device.

## Results

**Fig. 2.**
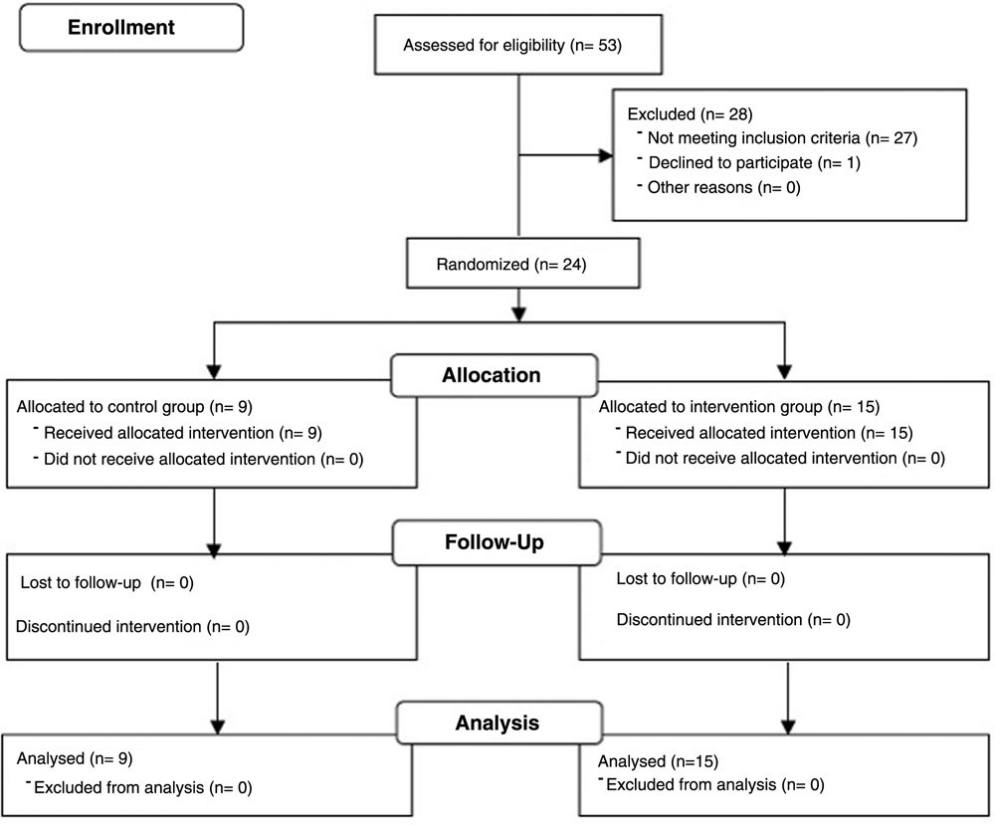
CONSORT diagram.

Fifty-three patients were assessed for eligibility, but 27 patients did not meet inclusion criteria primarily due to lack of Wi-Fi in the home and one declined to participate. The final sample included 24 patients who were randomly assigned to two study groups: (1) 9 controls and (2) 15 in the intervention group. Of patients enrolled, all completed the study. None were lost to follow-up (*Fig. 2*). See *Table 2* for patient characteristics.

**Table 2.** Patient Characteristics

	CONTROL GROUP (*n* = 9)	INTERVENTION GROUP (*n* = 15)	*p*
Female (%, *n*)	66.67 (6)	46.67 (7)	0.30
Age (mean in years)	9.32 ± 6.67	8.81 ± 5.74	0.85
Non-White (%, *n*)	77.78 (7)	46.67 (7)	0.14
Public insurance (%, *n*)	77.78 (7)	73.33 (11)	0.60
Tracheostomy alone (%, *n*)	11.11 (1)	13.33 (2)	0.69
Tracheostomy with ventilator dependence (%, *n*)	33.33 (3)	33.33 (5)	0.67
No. of diagnoses (mean)	11 ± 3.71	10 ± 3.40	0.64
No. of specialists (mean)	5.55 ± 1.87	5.87 ± 1.51	0.98

Encounter data are reported in *Table 3* and include information obtained from clinician surveys. The telehealth device was desired in 85 encounters. In the control group, 12 visits would have used the telehealth device if it were available, but occurred by telephone (92%) or electronic message (8%). In the intervention group, telehealth visits were attempted in 73 encounters. Most visits (79%) were scheduled. The most common complaint resulting in desire for a telehealth visit was respiratory/ear–nose–throat related.

**Table 3.** Encounter Data for Cases When a Telehealth Visit Was Desired

VARIABLE	CONTROL GROUP (*n* = 5, 12 ENCOUNTERS)	INTERVENTION GROUP (*n* = 15, 73 ENCOUNTERS)	*p*^a^
Type of encounter			0.000
Phone	11 (92%)	2 (3%)	
Electronic message	1 (8%)		
Scheduled telehealth visit		58 (79%)	
Unscheduled telehealth visit		13 (18%)	
Who did the clinician interact with			0.006
Clinician/legal guardian	7 (64%)	67 (92%)	
Home health nurse	3 (27%)	4 (5%)	
Other	2 (18%)	2 (3%)	
Chief concern for the encounter			0.008
Respiratory/ENT	4 (33%)	34/43 (79%)^b^	
Fever or acute illness symptoms	1 (8%)	4/43 (9%)^b^	
Seizure or neurologic symptoms	0	1/43 (2%)^b^	
Medical technology or equipment	1 (8%)	4/43 (9%)^b^	
Other	7 (58%)	8/43 (19%)^b^	
Practice visit + clinical question	0	5/73 (7%)	
Practice visit only		30/73 (41%)	

^a^ Chi-square analysis.

^b^ Adjusted to account for those encounters that were for practice visit only.

ENT, ear–nose–throat.

### Feasibility and Usability of the Telehealth Device in the Home

In the observation period, device connection was successful in 92% of attempted encounters. In 18% of attempts, connection was established with some reported difficulty, including device pairing issues or the video “freezing” during the encounter (*Table 4*). In four encounters, the telehealth device failed to connect, so usability data were based on 69 visits. In >92% of uses, ease of use, image and sound quality were acceptable (*Table 5*). Providers used the otoscope the least of all device elements, and had limited success with examinations due to cerumen buildup or caregiver discomfort. Clinicians were able to develop a clinical plan in 97% of visits based on available telemedicine data.

**Table 4.** Feasibility of Connectivity

	STUDY TIME PERIOD	
	PREMODIFICATION MONTH, *n* (%)	OBSERVATION MONTHS, *n* (%)
Not able to get a connection	0/23 (0)	4/50 (8.0)
Connection obtained but was problematic	15/23 (65.2)	9/50 (18.0)
Connection worked great	8/23 (34.8)	37/50 (74.0)

**Table 5.** Device Usability During Study

	STUDY TIME PERIOD	
USABILITY OF DEVICE ELEMENTS	PREMODIFICATION MONTH, *n* (%)	OBSERVATION MONTHS, *n* (%)
Percentage of visits used videoconferencing	23/23 (100)	46/46 (100)
Videoconferencing IMAGE quality acceptable	23/23 (100)	46/46 (100)
Videoconferencing SOUND quality acceptable	23/23 (100)	45/46 (97.8)
Videoconferencing EASE OF USE acceptable	23/23 (100)	46/46 (100)
Percentage of visits used stethoscope	18/23 (78.2)	44/46 (98.0)
Visits able to complete the examination using the stethoscope	18/18 (100)	42/44 (95.4)
Stethoscope used for heart sounds	12/18 (66.7)	17/44 (38.6)
Stethoscope used for breath sounds	18/18 (100)	44/44 (100)
Stethoscope SOUND quality acceptable	18/18 (100)	43/44 (97.7)
Percentage of visits used thermometer	19/23 (82.6)	36/46 (78.0)
Thermometer readings believable	19/19 (100)	34/36 (94.4)
Percentage of visits used camera	17/23 (73.9)	24/46 (52.0)
Visits able to complete the examination using the camera	16/17 (94.1)	24/26 (92.3)
Camera used for skin assessment	8/17 (47.0)	3/24 (12.5)
Camera used for oropharyngeal examination	9/17 (52.9)	22/24 (91.7)
Camera used for other (WOB assessment)	2/17 (11.8)	0/24 (0)
Camera IMAGE quality: acceptable	17/17 (100)	24/26 (92.3)
Percentage of visits used otoscope	13/23 (56.5)	23/46 (50.0)
Visits able to complete the examination using the otoscope	7/14 (50)	16/25 (64.0)
Otoscope IMAGE quality acceptable	13/14 (92.8)	24/25 (96.0)

WOB, work of breathing.

## Impact of the Telehealth Device in the Home

### Resource Utilization

**Fig. 3.**
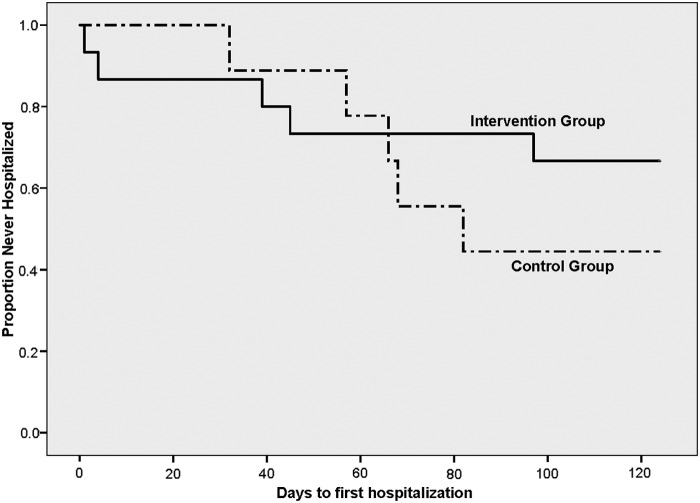
Kaplan–Meier survival analysis demonstrating time to first hospitalization by study group.

*Table 6* shows resource utilization by group for the premodification month and observation period. In the intervention group, five patients required nine hospitalizations. In the control group, five patients accounted for six hospitalizations. The ICU hospitalization rate was lower for the intervention group compared with the control group (0.77 vs. 1.14). Similarly, the pediatric floor hospitalization rate was lower for the intervention group compared with the control group (0.32 vs. 0.67). The rate of ED visits and acute office visits was higher for the intervention group than for the control group. Length of hospitalization as tested with a negative binomial regression showed that the control group had a higher occurrence of events though the difference was not statistically significant (Beta 0.69, Wald 95% confidence interval −0.14 to 1.5, *p* = 0.10). Using Kaplan–Meier survival analysis, 67% of the intervention group remained out of the hospital compared with 44% in the control group (*Fig. 3*).

**Table 6.** Resource Utilization and Costs

	CONTROL GROUP				INTERVENTION GROUP			
	NO. OF VISITS	DIRECT COST, $	VISIT RATE (COUNT/GROUP *n* × MONTHS)	COST RATE (COSTS FOR VISITS × VISIT RATE), $	NO. OF VISITS	DIRECT COST, $	VISIT RATE (COUNT/GROUP *n* × MONTHS)	COST RATE (COSTS FOR VISITS × VISIT RATE), $
Acute office visit	5	920.00	0.139	127.88	10	1,840.00	0.167	307.28
Premodification month					4			
Observation months	5	920.00	0.139	127.88	6	1,104.00	0.100	110.40
Telehealth visit					43	7,912.00	0.717	131.93
Premodification month					15			
Observation months					28	5,152.00	0.467	85.93
ED visit	2	3,926.00	0.056	219.86	7	13,741.00	0.117	1,607.70
Premodification month					1			
Observation months	2	3,926.00	0.056	219.86	6	11,778.00	0.100	1,177.80
Hospitalization days-floor	24	43,896.00	0.667	29,278.63	19	34,752.00	0.317	11,016.38
Premodification month					1			
Observation months	24	43,896.00	0.667	29,278.63	18	32,923.00	0.300	9,876.90
Hospitalization days-ICU	41	101,229.00	1.139	115,299.83	46	113,574.00	0.767	87,111.26
Premodification month					9			
Observation months	41	101,229.00	1.139	115,299.83	37	91,353.00	0.617	56,364.80
Total costs without telehealth visits		149,971.00		144,926.20		163,907.00		100,042.62
Total costs with telehealth visits						171,819.00		100,174.55

All costs quoted in US dollars.

ICU, intensive care unit.

### Clinician and Family Satisfaction

Clinicians reported being very satisfied with the use of the telehealth device during encounters (median 4.0, IQR 4.0–4.0) and noted specific benefits, including the ability to provide detailed instructions (e.g., gastrostomy tube reinsertion), in-home care for posthospitalization visits, specialist consultation, and scheduled outpatient follow-up visits. During one telehealth visit, critical hypoventilation was detected and emergency response initiated. Throughout the study period, caregivers (*n* = 13) reported being very satisfied (median 4.0, IQR 3.5–4.0) and very comfortable (median 4.0, IQR 3.5–4.0) with the use of the telehealth device, rating it very easy to use (median 3.0, IQR 3.0–4.0). Caregiver comments suggested that comfort with the device improved over the study period; however, survey response scores did not demonstrate a significant change over time (first use median 3.5, IQR 3.0–4.0, *n* = 10; last use median 3.0, IQR 3.0–4.0, *n* = 7). One parent stated that the ability to interact with the clinician through telehealth reduced her worries about staying at home. Caregivers from both groups rated interactions with clinicians and the complex care program satisfaction similarly (control group median 4.0, IQR 3.7–4.0; intervention group median 4.0, IQR 4.0–4.0).

### Costs

Direct costs and cost rates (*Table 6*) were estimated per encounter type for each group over the complete 4-month study period. Calculated cost rates (direct cost of encounter multiplied by the visit rate) showed a $44,751.65/USD ($9,425/USD per patient) cost savings for the intervention group. While the intervention group had a greater number of acute office and telehealth visits, the intervention group had a lower number of hospitalization days, which contributed to the overall lower cost rate.

There were seven telehealth visits that the investigators recorded as preventing an in-person visit, including three ED visits, three outpatient visits, and an ICU hospitalization. The ICU hospitalization that was prevented would typically have resulted in a 3-week ICU stay based on previous experience. Based on our direct cost for the ED visits, acute outpatient visits, and 21 days in the ICU, the prevented services resulted in ∼$58,300/USD in potential direct cost savings.

## Discussion

Telehealth to facilitate evaluation of children in both inpatient and outpatient settings has been shown to be a safe and effective means of providing care within the patient-centered medical home.^15–18^ For children with special health care needs, telehealth solutions can help meet a critical need. This has been explored in both school-based and home-based videoconferencing solutions as well as pediatric specialist visits with reported success.^13,19,20^ To our knowledge, this is the first study to explore the home use of peripheral devices, particularly those used by caregivers, to facilitate telehealth visits for CMC.

Overall, our study demonstrated that the telehealth device was feasible to use by caregivers in the home. Our study was conducted in the suburbs of a large metropolitan area, and the device and software bandwidth needs were well under the Federal Communications Commission's established benchmark of 25 Mbps download/3 Mbps upload speed.^21^ Despite this, some homes required supplemental devices to provide an adequate signal for the telehealth device, though the exact causes for connectivity difficulties were not identified.

The study required modification after attempted visits in the initial month encountered connectivity and device pairing issues. As this was the first in-home trial of the telehealth device, software modifications were necessary but easily made. Although the study data focused on the planned 3-month period, the premodification data were included to illustrate the potential issues that could be encountered in using a device with patients or caregivers in the home, and the need for a testing and training period to identify issues related to the device and/or home connectivity.

Caregivers reported that the device was easy to use throughout the study. The premodification month and scheduled practice visits allowed both caregivers and clinicians to become more familiar with the device and basic troubleshooting. While an entire practice month may not be practical, or even necessary, some degree of practice and use in nonurgent situations is likely to be of benefit in future programs. The finding that caregivers sometimes identified issues not previously relayed to the care team during practice visits demonstrates the value of adding video and peripherals to regular communications, helping us to facilitate earlier detection of clinical issues as well as strengthening the patient–clinician relationship.

In other telemedicine studies, examinations are rated as equivalent or sometimes superior to in-person physical examination.^8–10^ In our study, clinicians noted that the auscultation component helped filter out background noise and provided high-quality sound for evaluation. Similarly, the oral examination device had a built-in tongue blade, allowing the caregiver to more easily perform an examination on a child who may not be able to follow directions to open his mouth. The least successful component was the ear examination, due to both patient factors such as cerumen buildup and caregiver lack of experience. Future interventions to help improve caregiver comfort with this component may deliver success.

Other telehealth studies have shown a decrease in hospital-based resource utilization.^20^ We also found this to be true with decreased rates of hospital floor and ICU days in the intervention group. While the rate of ED visits was higher in the intervention group, a review of all ED referrals showed that regardless of group and use of telehealth, the patient's condition warranted an ED visit. Interestingly, some telehealth visits revealed urgent issues that would not have been detected by phone and led to appropriate ED referrals. The intervention group also had a higher percentage of patients not hospitalized during the study period, which suggests that the addition of telehealth visits was effective and led to lower cost rates. In addition, the use of the telehealth device may have prevented more costly resource utilization by referring for a timely ED visit rather than a long-term hospitalization. As health care shifts to more value-based care, these savings are important in weighing program overhead costs and determining return on investment. Estimated cost savings may have been higher taking into account cost of transportation and gas, lost caregiver wages, and childcare fees for other household children.^20^ There are also intangible benefits to families with a CMC related to decreased anxiety from the knowledge that caregivers can connect with their medical home directly and easily using telehealth.

### Limitations

The results of this study are highly promising in evaluating the benefits of telehealth in the home setting of CMC, but are limited by the small sample size and brief study period. Of the clinic population evaluated, 50% did not meet eligibility criteria due to lack of home WiFi and/or language barriers. This could be partially addressed in future studies with the use of technology, which would allow multipoint calls for language services. Supplemental home Wi-Fi devices may address some access issues, although access to broadband remains a challenge in certain geographic areas. In addition, certain factors included in this study may not be feasible in practice. The cost in time, personnel, and equipment for individual home setup visits, supplementing home connectivity, regular practice visits, and providing accessory devices must be weighed against the benefits of successful connections. Practice visits were included in this study to help with testing and training related to the home technology. Since these were conducted by clinicians, in some cases, an unplanned clinical visit resulted. Although this represents a small number of visits, there is a potential impact of increased contact by clinicians with this subset of interactions. Regardless of group, caregivers had equal access to clinicians by phone and electronic message. As the primary purpose of this study was to evaluate the feasibility and usability of the device, future studies are needed to more fully assess the impact of various types of connections for targeted patient populations and circumstances.

## Conclusion

This study demonstrated successful use of a telehealth device by a caregiver in the homes of children with complex medical conditions. Caregivers were comfortable with and satisfied using the device. Clinicians found the device useful in gathering data to inform patients' plans of care. Compared with the control group, those with access to telemedicine had fewer hospital days and reduced cost rates. This study provides a foundation for examining the value of telehealth in the homes of other populations with special health care needs.

## Footnotes

^*^ Identifier: NCT02849938, https://clinicaltrials.gov/show/NCT02849938
